# Development Trend in Composition Optimization, Microstructure Manipulation, and Strengthening Methods of Die Steels under Lightweight and Integrated Die Casting

**DOI:** 10.3390/ma16186235

**Published:** 2023-09-15

**Authors:** Ze-Ju Bao, Hong-Yu Yang, Bai-Xin Dong, Fang Chang, Chuan-De Li, Ying Jiang, Liang-Yu Chen, Shi-Li Shu, Qi-Chuan Jiang, Feng Qiu

**Affiliations:** 1 State Key Laboratory of Automotive Simulation and Control, Jilin University, Changchun 130025, China; baozj22@mails.jlu.edu.cn (Z.-J.B.); dongbx20@mails.jlu.edu.cn (B.-X.D.); changfang@jlu.edu.cn (F.C.); licd21@mails.jlu.edu.cn (C.-D.L.); jiangying22@mails.jlu.edu.cn (Y.J.); jiangqc@jlu.edu.cn (Q.-C.J.); 2 Key Laboratory of Automobile Materials, Ministry of Education and Department of Materials Science and Engineering, Jilin University, Renmin Street No. 5988, Changchun 130025, China; 3 School of Materials Science and Engineering, Jiangsu University of Science and Technology, Zhenjiang 212003, China; lychen@just.edu.cn; 4 School of Mechanical and Aerospace Engineering, Jilin University, Renmin Street No. 5988, Changchun 130025, China

**Keywords:** automobile lightweight, integrated die casting, die steel, traditional strengthening processes, nanoparticles strengthening process

## Abstract

In the general environment of lightweight automobiles, the integrated die-casting technology proposed by Tesla has become the general mode to better achieve weight reduction in automobiles. The die-casting mold required by integrated die-casting technology has the characteristics of large scale and complexity. Hence, higher requirements are put forward for the comprehensive performance of the die steel. Despite the stagnation in the progress of conventional strengthening methods, enhancing the performance of die steel has become increasingly challenging. Indeed, it necessitates exploring novel die steel and optimizing heat treatment and reinforcement technologies. This article summarizes and analyzes the development status of die steel and corresponding heat treatment and microstructure manipulation as well as strengthening methods and elaborates on an excellent nano-strengthening technology. Furthermore, this review will aid researchers in establishing a comprehensive understanding of the development status of die steel and the processes utilized for its strengthening. It will also assist them in developing die steel with improved comprehensive performance to meet the high demand for mold steel in the integrated die-casting technology of the new era.

## 1. Introduction

**Figure 1:**
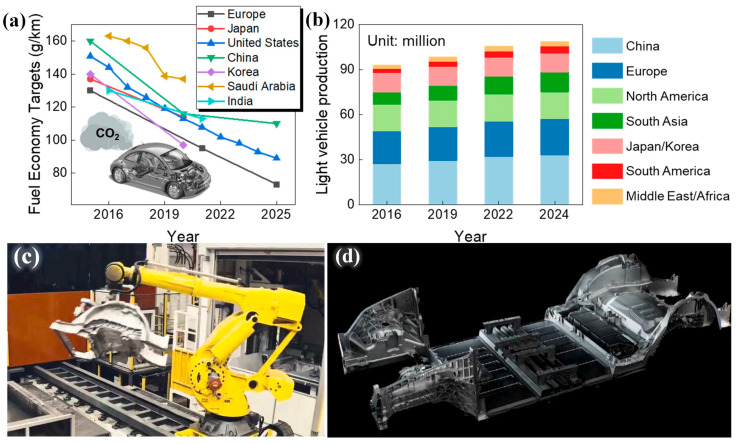
Urgent needs for lightweight vehicles: (**a**) fuel economy targets (average CO_2_ emissions per kilometer drive) in different countries (passenger vehicles) and (**b**) light vehicle production in major markets [3]. Integrated die-casting of Tesla Model Y: (**c**) rear floor structure and (**d**) schematic diagram of car body assembly [12].

In its pursuit of slowing down global warming and achieving sustainable development, the automotive industry has confronted several challenges in recent years. These challenges include the steady elevation of carbon emission standards in different countries, as depicted in Figure 1a. Residents are facing additional concerns, such as the escalating gasoline prices, while manufacturers are grappling with the mounting production costs of new energy vehicles [1,2,3]. Driven by these factors, the automotive industry urgently needs new technological directions to reduce consumption and save energy. Therefore, the lightweight concept has been introduced in the automotive field, which puts forward the integrated die-casting technology. As shown in Figure 1b, the output of light vehicles in various countries increases yearly. The lightweight automobile is the trend in automobile development in the world and it aims to achieve energy saving and emission reduction.

Currently, three main directions for the lightweighting of automobiles are encountered: lightweighting of materials, lightweighting of structures, and lightweighting of manufacturing processes [4,5,6,7,8]. The manifestation of lightweight materials is to replace materials with higher specific gravity with lightweight materials. As such, the curb weight of automobiles is reduced. Aluminum alloy is currently the most widely used lightweight material. Structural lightweighting optimizes the structure, size, shape, and morphology of parts to obtain the best design parameters, thereby reducing the amount of material used. The lightweighting aim of the manufacturing process is to achieve the improvement of material performance, shape, and morphology by optimizing the process; the integrated die-casting technology is an extremely important part of lightweight manufacturing. The integrated die-casting realizes the integral casting of aluminum alloy parts and replaces many scattered parts with a large integral part, reducing the number and equipment of parts. Preparing light, complex, and large-scale aluminum alloy parts is conducive to promoting the lightweight process. Incorporating lightweight and integrated die-casting techniques in automobile manufacturing proves highly effective in addressing the challenges mentioned above, consequently serving as a crucial means to achieve energy efficiency and emission reduction [9,10].

Automotive lightweighting refers to reducing the curb weight of a vehicle under the condition of ensuring strength and safety to reduce energy consumption. At present, automotive lightweighting has become a major trend in the global development of automobiles [11]. The integrated die-casting technology was first proposed by Tesla. It can highly integrate multiple scattered parts into one casting part. One-step molding eliminates the complex process of split stamping followed by welding and assembly and it also greatly reduces manufacturing costs and allows for the fabrication of products with more complex structures. Tesla began applying the one-step molding to the Model Y body-in-white rear underbody in 2020. Figure 1c,d entails schematic diagrams of the rear floor structure and body assembly of Model Y, respectively. Compared with the traditional process, one-step molding has saved manufacturing costs and achieved remarkable results [12]. Various countries are actively following up on integrated die-casting and the die-casting process has become a disruptive technology in the automobile manufacturing process [3]. The die-casting machine is expected to replace the welding robot as the significant equipment in the automobile manufacturing field, thereby upgrading the manufacturing method of automobiles.

Currently, most lightweight materials used in the automotive industry are aluminum alloy materials and magnesium alloy materials [3,13,14]. The manufacturing process of aluminum alloy and magnesium alloy die-casting parts requires molds [15,16,17,18,19]. The integrated die-casting mold is large in weight and volume for large-tonnage integrated die-casting parts. Compared to conventional aluminum alloy molds, the structure of advanced molds is more intricate, demanding stricter precision and quality requirements. Moreover, some problems may be caused by large-scale die-casting molds. For example, alloy element segregation may occur in the large-size ingot for preparing large-size molds. Hence, larger dies may encounter issues such as uneven microstructure and composition during the forging process, leading to significant performance discrepancies between the central and peripheral regions of the die. These problems would cause damage to the quality and life of the integrated die-casting molds. Therefore, manufacturing integrated die-casting molds is more difficult and there are still high barriers. Under the trend in lightweight and integrated die-casting, higher requirements are put forward for die steel. This article systematically reviews the present development status and emerging trends in integrated die-casting die steels. It comprehensively summarizes and offers insights into prospects, aiming to inspire upcoming researchers to build upon current studies and forge new pathways to enhance the performance of die steels.

## 2. Development in Composition Optimization of Die Steel for Integrated Die Casting

Die steel is an extremely important part of modern industry. With the continuous penetration of lightweight and integrated die-casting in the automotive industry, the demand for hot-work die steel continues to increase and countries all over the world attach great importance to its development. And in the international environment of lightweight and integration, molds tend to be large, complex, and precise, which poses a huge challenge to the comprehensive performance of mold steel. As such, the development and application of die steel with enhanced performance have become imminent necessities [20,21,22].

### 2.1. Traditional Hot-Working Die Steels

Due to the need for environmental protection and energy saving, the lightweighting of automobiles has become the trend in automobile development in the world. Integrated die-casting will be an important direction for developing lightweight automobiles now and in the future. Most of the mold materials used in die-casting are hot-working molds. Therefore, developing hot-working die steel with better performance can lay a good foundation for developing integrated die casting. Table 1 lists the traditional hot-working die steel grades commonly used worldwide. However, a higher level of hot-working die steel has been proposed under the new era of integrated die casting that Tesla has opened. In order to satisfy the demands of large-scale and intricate die-casting components, it is imperative to enhance the comprehensive performance of die steel. Such an objective involves improving its hardenability, thermal stability, thermal conductivity, and toughness while prolonging its service life. The traditional hot-working die steel cannot meet the requirements of making large-scale molds regarding hardenability. Therefore, developing new hot-working die steels with better properties is very necessary and meaningful.

### 2.2. New Hot-Working Die Steels

There are two main research directions for hot-working die steel. One is to optimize the compositions of die steels [23,24,25,26]. Most of them are developing towards low carbon and low alloying elements. Reducing the carbon content can improve the hardenability of die steel. To modify the characteristics of hot-working die steels, it is recommended to decrease the use of typical alloying elements (such as C, Cr, Si, and V) and introduce trace elements like Ni, Al, and Nb [27,28,29]. Another approach is to enhance the production process and heat treatment technology employed in die steel manufacturing [30,31,32].

**Figure 2:**
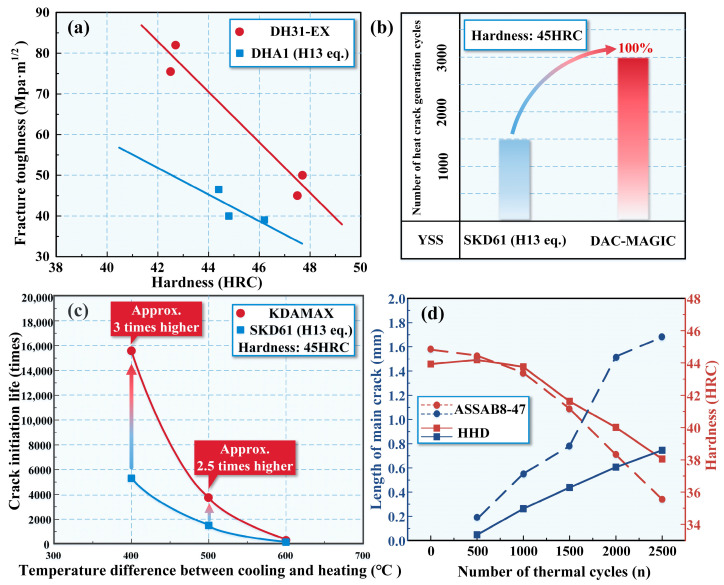
Performance comparison diagram of different steels: (**a**) Fracture toughness [33]. (**b**) Number of heat crack generation cycles [34]. (**c**) Crack initiation life [35]. (**d**) Length of main crack and hardness [42].

In recent years, the development in new hot-working die steel is mostly carried out based on steel grades aligned with H13 steel, such as DH31-EX steel of Japan Daido Industry Co., Ltd. (Nagoya, Japan); DAC-MAGIC steel of Japan Hitachi Metals Co., Ltd. (Tokyo, Japan); and KDAMAX steel of Japan High Frequency Steel Co., Ltd. (Tokyo, Japan). All of them are new high-performance hot-working die steels [33,34,35]. Figure 2 depicts a performance comparison diagram highlighting significant enhancements. DH31-EX steel exhibits exceptional hardenability and offers high toughness in the core region of large molds (Figure 2a). As a result, it effectively minimizes the risk of significant mold cracking during usage, consequently enhancing the overall lifespan of the molds. It is suitable for large die-casting molds and forging molds. The high-temperature strength of DAC-MAGIC steel is extremely high, along with outstanding resistance to heat cracks produced when crystallizing at high temperatures (Figure 2b). DAC-MAGIC steel demonstrates a heat crack generation cycle that is twice as long as SKD61 and has a lower production cost. However, it should be noted that its fatigue properties are weaker compared to DH31-EX steel. KDAMAX steel, employed in Al and Zn die-casting molds, currently boasts the highest strength-to-toughness ratio among the steels offered by Japan High Frequency Steel Co., Ltd. (Figure 2c). It exhibits commendable thermal crack resistance, exceptional resistance against crack propagation at stress-concentrated mold corners, and remarkable resistance to significant cracks originating from water holes. Consequently, it is ideally suited for hot forging dies and molds featuring intricate shapes, including projections and depressions.

The new Dievar hot-working die steel from ASSAB Sweden is a premium steel grade developed specifically for the demanding applications of high-pressure die casting [36,37]. The die steel sets the industry benchmark for its superior toughness and ductility and the longest die life with a low risk of cracking. As a result of the changes in the automotive industry, which have placed higher demands on larger and more complex parts, the company has adopted the latest remelt technology to further improve homogeneity and purity for die steel. Furthermore, process innovations and enhancements have been implemented across the production line. The newly generated Dievar die steel has increased its toughness to a new level, making it extremely suitable for making large dies. Dievar die steel has excellent resistance to thermal cracking, early and integral cracking, thermal wear, and plastic deformation. Moreover, Dievar die steel has better high-temperature properties than H13 and H11 steels [38]. The newly developed Dievar steel is a good choice for demanding die casting, forging, and extrusion dies.

In the new era, the surface quality of automobiles holds increasing significance, particularly for structural or electric vehicle components that entail stricter requirements. Consequently, molds are inclined to become more intricate and precise. The CS1 new high-quality hot-working die steel developed by Kind and Co Germany can meet extremely high surface quality requirements and narrow shape tolerances. It also has good toughness and high hardness of up to 58 HRC [39]. CS1, a new hot-working die steel, is the realization of the perfect combination of toughness and thermal shock resistance, which is very suitable for making large precision molds with complex geometry or strict shape tolerance.

The group Jiang from Jilin University in China and China FAW Group Co., Ltd. (Changchun, China), jointly developed a new high-performance hot-working die steel: HHD steel [40]. This innovative and fully martensitic hot-working die steel disrupts the traditional design principles of international advanced hot-working die steel, which typically features medium to low Cr content. Instead, it raises the Cr content to approximately 10%, marking a breakthrough in design concepts. The high content of Cr gives it extremely excellent hardenability and oxidation resistance. An appropriate amount of nitrogen is also added to improve the thermal strength of the material by the solid solution strengthening of nitrogen and the precipitation strengthening of carbon and nitride [41]. In addition, a new strengthening and toughening heat treatment process is also designed and optimized so that many nano-sized carbides are dispersed and distributed in the martensite matrix. The performance of HHD steel is further improved. By employing novel alloy composition design, microalloying techniques, and advanced methods for strengthening and toughening heat treatment, a state-of-the-art high Cr hot-working die steel (HHD steel) has been successfully developed. This steel possesses exceptional properties such as superior thermal and mechanical fatigue resistance, high hardenability, high-temperature wear and oxidation, and high-temperature creep. As a result, its service life has been significantly enhanced. The high-temperature properties of HHD steel are higher than those of the H13 series of hot-working die steels. Figure 2d compares the high-temperature properties of HHD steel and ASSAB8407 steel [42]. When subjected to an equal number of thermal cycles, the main crack length of HHD steel is considerably shorter than that of 8407 steel. This observation suggests a substantial improvement in its thermal fatigue resistance.

Additionally, the HHD steel exhibits slower changes in hardness compared to that of 8407 steel during the thermal cycling process. It is also interesting to note that the HHD steel slightly increases hardness after 500 thermal cycles. Therefore, this new high Cr hot-working mold steel has an excellent performance in making large aluminum alloy die-casting molds and has good economic and development prospects.

As mentioned above, the die steels have outstanding performance, characteristics, and advantages in making integrated die-casting molds, which can well meet the requirements of large-scale and complex parts. The alloy compositions of these novel hot-working die steels have been optimized to enhance their overall properties. Such an outcome is particularly exemplified by the noteworthy increase in the Cr content of HHD steel, which has paved the way for a fresh approach to optimizing alloy compositions. Optimization of the composition of the die steel is a very important strengthening method. Optimizing the production process and heat treatment technology is another effective way of enhancing the properties of hot-working die steel as it greatly influences the enhancement of its microstructure and overall performance. The following section will provide an overview of the current development in the production and heat treatment procedures employed in hot-working die steel.

## 3. Development in Microstructure Manipulation and Strengthening Methods of Die Steel for Integrated Die Casting

The importance of developing new high-performance hot-working die steel is beyond doubt. By doing so, it becomes possible to significantly enhance the performances of die steels, reduce raw material costs, and concurrently improve performance while considering material efficiency. However, developing new die steel requires a certain level of selectivity and the widespread application of new mold steel is still limited in most countries, with certain promotional restrictions. Another widely adopted approach that substantially improves the production process and heat treatment technology of die steel is prevalent and has proven to be highly effective.

### 3.1. Melt Purification and Impurity Removal

**Figure 3:**
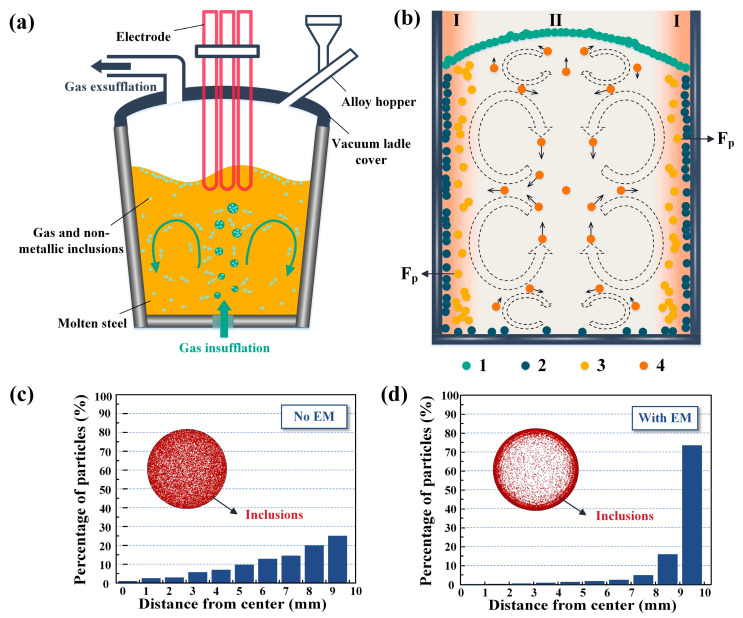
Schematic diagram of different melt purification processes: (**a**) Schematic diagram of air-blowing purification [49]. (**b**) Schematic diagram of electromagnetic (EM) separation. Distribution and apparent percentage of horizontal inclusions in steel: (**c**) No EM. (**d**) With EM [58].

The primary steels contain a lot of gas and inclusions during the smelting process, significantly affecting their performance and life [43,44,45]. Therefore, removing impurities and purifying the molten steel is necessary. The traditional melt purification process mainly includes the solvent purification method, Ar-blowing purification method, filtration purification method, sedimentation purification method, and composite purification method, among which the Ar-blowing purification method has a simple process, cheap equipment, and remarkable purification effect. Ar-blowing purification is a current method commonly used in industry and it is the most important method for purifying molten steel [46,47,48]. The principle of Ar-blowing purification is shown in Figure 3a [49]. It entails injecting tiny bubbles of Ar gas into the molten steel by permeable bricks, ensuring that argon, which does not partake in any metallurgical reactions, is predominantly used. Each of these Ar bubbles acts as a miniature vacuum within the steel. As a result of the partial pressure difference, the bubbles effectively transport gases such as H, N, and O, along with non-metallic inclusions, within the molten metal. These bubbles rise and eliminate the impurities above as they reach the surface. Numerous fine and dispersed air bubbles can also continuously drive the molten steel to tumble, which can achieve a better removal effect and make the composition and temperature of the molten steel uniform. This process can effectively reduce the content of harmful gases and remove non-metallic inclusions in molten steels. So far, the Ar-blowing purification process has attained a high degree of maturity and has found wide application in various industries. It plays a vital and indispensable role in traditional melt purification methods. However, with the constantly growing demands for steel quality, there arises a need for even stricter standards regarding the inclusion of content in high-quality steel.

The properties and quality of steels are related to the number, type, shape, size, and distribution of non-metallic inclusions [50]. The existence of non-metallic inclusions in steel can disrupt the continuity and integrity of the matrix, leading to reduced plasticity, toughness, and fatigue resistance of the material [51,52,53]. In some cases, the non-metallic inclusions will also cause cracks in the steel during heat treatment or even break during use. However, these inclusions are unavoidable and cannot be completely removed. Therefore, the effective removal size range of non-metallic inclusions becomes an important criterion for judging the purification ability of the method. Although the traditional melt purification method in the industry can remove most large-size impurities, the removal effect is not expected for inclusions with a size smaller than 20 μm.

Enrico et al. [44] optimized the foam ceramic filter using nanomaterial composite filter coating. The ceramic filter can achieve a good filtering effect on large non-metallic inclusions, while the removal rate of inclusions smaller than 10 μm is lower than 20%. Consequently, Enrico et al. [44] functionalized filter materials based on the idea of “active” and “reactive” coating proposed by Aneziris et al. [54], selecting different types of nanoparticles as new coatings for foam ceramic filters. Through the research, it has been discovered that Al_2_O_3_ can be efficiently filtered, with the GO-C filter showcasing the most effective removal of Al_2_O_3_. This results in a significant reduction in the content of non-metallic inclusions within the melt. However, this method also has certain limitations. It can only effectively filter a single non-metallic inclusion and be applied to some production processes with specific needs. Alongside the size inclusions, larger inclusions are also generated, necessitating the use of the filter in combination with other filters rather than relying on it alone. Moreover, the manufacturing process of the nano-coating foam ceramic filter is also cumbersome. These problems limit the wide application of this filter.

The traditional melt purification methods have almost fallen into a bottleneck. Therefore, a new impurity removal process with an external physical field has been developed, achieving remarkable results in removing inclusions [55]. In recent years, electromagnetic (EM) separation technology has been regarded as an emerging technology for metal purification [56]. This technology capitalizes on the disparity in electrical conductivity between the inclusions and the melt, leveraging EM force to segregate the two entities. It can even efficiently separate inclusions as small as 1–2 μm [57]. At present, EM separation technology can purify many metallic melts. A feasible method for effectively separating inclusions in molten steel is high-frequency EM separation [58]. Figure 3b is a schematic diagram of the principle of EM separation. Due to the EM force difference between Region I and Region II, most inclusions in Region II are brought to Region I along with the melt flow. The inclusions in molten steel have four different ends (1, 2, 3, and 4 in Figure 3b). In the middle of zone II, only a small quantity of inclusions persist, while the majority of inclusions congregate together, facilitating their subsequent effortless removal. Figure 3c,d shows the particle distribution of inclusions in the horizontal direction before and after applying EM separation, respectively. It is not difficult to see that the use of high-frequency EM separation in molten steel can effectively concentrate and wrap the inclusions on the inner wall of the crucible to achieve significant purification of molten steel, thereby improving the properties of the material [58].

Compared with traditional separation methods, EM separation technology results in no pollution to the environment or molten metal and is highly efficient and easy to control. By adjusting the parameters of the electric and magnetic fields, the magnitude and direction of the particle separation force can be changed. EM separation also has a good separation effect on inclusions with smaller sizes and is not limited to a single type of inclusions. Despite the numerous advantages of this technology, the overall cost of the separation process is relatively high compared to alternative methods such as conventional filtration devices, bubble flotation, and centrifugation. The economic viability significantly influences the application scope of this technology and further innovations and enhancements are necessary to fully integrate it into industrial production. The current state of melt purification is characterized by traditional methods being constrained by a bottleneck, making introducing innovations with substantially improved results challenging. Although EM separation technology has outstanding effects, simple operation, and no pollution, it still has a long way to go before it can be applied to industry.

### 3.2. Optimal Treatment of Microalloying

Alloying hot-working die steel is an important way to improve its service life. The performance of hot-working die steel can be influenced to varying degrees by different alloying elements and their respective content levels, resulting in either positive or negative effects [27,59,60,61,62,63,64,65]. Table 2 lists the appropriate content ranges of some alloying elements and their functions in hot-working die steels. C is an important constituent of steel whose content affects the hardness, hardenability, strength, and toughness of die steel. C can strengthen the steel by solid solution and precipitation strengthening (by forming carbides) [66,67]. The formed carbides should be dispersed and precipitated in hot-working die steel during the tempering process to produce secondary hardening. Therefore, the carbon content of most hot-working die steels is kept in the range of 0.3~0.6%. A small amount of carbides can be formed and the hardenability and toughness of the die steel can be guaranteed. Elevated C content can greatly enhance the strength of die steel; however, excessively high C content may lead to reduced toughness and plasticity, ultimately impacting its durability. During the alloying design process, it is important to minimize the C content to ensure optimal strength. Cr is the most important alloying element in die steel and an alloying element commonly contained in die steel [40]. The Cr content in hot-working die steel is usually 2% to 12% and most hot-working die steels contain two series of 3% and 5% Cr. Cr can enhance the hardness, strength, wear resistance, high-temperature oxidation resistance, high-temperature mechanical properties, and hardenability of steels. However, when the Cr content in the die steel is high, the austenite grains tend to be coarse. During the solidification process, an excessive amount of Cr tends to segregate, leading to the precipitation of primary carbides in the final stages of solidification. These primary carbides are difficult to eliminate during subsequent deformation and heat treatment. Therefore, excessive Cr content will lead to a declined performance of the die steel.

Mo can precipitate from martensite in Mo_2_C during the tempering process, serving as a secondary hardening alloying element. In order to achieve the desired secondary hardening effect, it is crucial to ensure that the Mo content reaches a specific threshold [68,69]. However, when the Mo content is too high, Mo_6_C will precipitate in the higher temperature region, resulting in the weakening and embrittlement of the steel. Studies have shown that the optimal and cost-efficient outcome is achieved by maintaining Mo content ranging from 2.0% and 2.5% [70]. The V element can play the role of grain refinement. For commonly used hot-working die steel, V is an important secondary hardening element [71,72]. V can also be combined with N to prevent or mitigate the strain aging caused by the presence of the N element. The tiny particles generated by the combination can also strengthen the steel. Due to the strong affinity between V and C, a slightly higher content of V will lead to a decrease in mechanical properties due to carbide aggregation, so the content of V in the die steel is kept at a low level.

In addition to the above alloying elements, many elements, such as Si, Mn, Nb, etc., can be alloyed for hot-working die steel to keep the content within the range of optimal steel performance [73,74]. Through continual exploration of alloy composition in hot-working die steel, the performance of die steel has been continuously enhanced, resulting in the development in successive series of hot-working die steel. This progression began with the low Cr-Mo series in the 1920s, followed by the H series in the 1970s, the QRO series in the 1990s, and finally, the new generation of hot-working die steel developed after 2000. The development in alloying treatments for hot-working die steel primarily revolves around achieving a low carbon and alloying content. The common approach often involves reducing carbon content while increasing the levels of Mo and Ni to enhance the hardenability of the steel. Additionally, it involves reducing the presence of commonly used alloying elements such as Si, Cr, and Mn while incorporating trace elements like Ni and Nb. This result improves the performance of hot-working die steel and fulfills the requirements of large-scale molds.

**Table 2:** The suitable content range and effect of some alloying elements in hot-working die steels.

Alloy Element	Content Range	Main Functions and Features	Ref.
C	0.3–0.6%	Increases the strength, hardness, and wear resistance of steel due to the formation of solid solutions and carbides	[66,69]
Cr	2.0–12.0%	Ubiquitous in die steel, improves wear resistance, corrosion resistance, toughness, hardenability, etc.	[40]
Mo	1.0–3.0%	The main alloying element for secondary hardening can also effectively improve the thermal strength of steel	[68,69,70]
V	0.05–1.2%	Refining grain and microstructure, improving tempering stability, and secondary hardening effect	[71,72]
Mn	0.15–1.0%	Improve the strength, hardness, and hardenability of steel without much damage to the plasticity and toughness	[73]
Si	≥0.4%	Replacing ferrite and solid solution strengthens effectively and improves the strength, hardness, and tempering stability of steel	[27]

**Figure 4:**
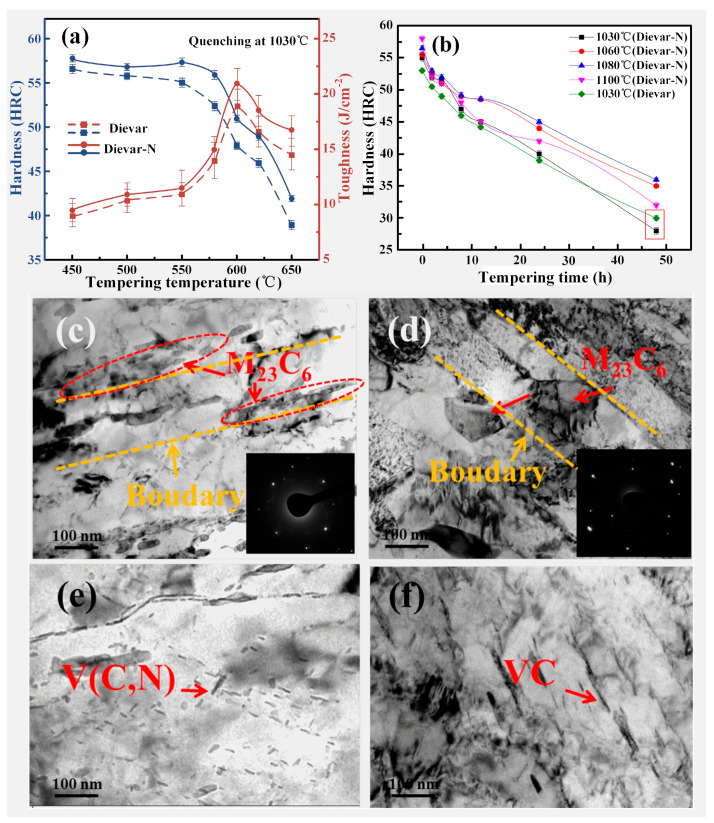
Effect of N on properties of hot-working die steel: (**a**) hardness and toughness of samples as a function of temper temperature [75] and (**b**) tempering stability of samples at different quenching temperatures. TEM micrographs of steel tempered at 600 °C (48 h): (**c**,**e**) are M_23_C_6_ and V.C. carbides in Dievar-N steel, respectively. (**d**,**f**) are M_23_C_6_ and V.C. carbides in Dievar steel, respectively [76].

For a considerable period, N was considered a harmful element in steel. However, it was not until the 1940s that people gradually began recognizing its potential benefits. N can improve the strength and corrosion resistance of austenitic stainless steel. However, there are few studies on N in hot-working die steel. Gu et al. [75] carried out trace N alloying of Cr-Mo-V series of hot-working die steel (Dievar steel). Figure 4a shows the trend in hardness and toughness of the two types of steels with the increase in tempering temperature. It can be seen that after adding a trace amount of N, the strength and toughness of the hot-working die steel are improved at different tempering temperatures; the comprehensive performance is the highest at the tempering temperature of 600 °C. The addition of trace N promotes the formation of V(C,N), a compound consisting of the elements vanadium, carbon, and nitrogen, thereby increasing the melting point and stability of the precipitates. Gu et al. [75] also investigated the strengthening mechanism of trace N alloying and found that the main strengthening mechanisms are grain refinement and precipitation strengthening. 

Gu et al. [76] also investigated the effect of N on the tempering stability of hot-working die steel and found that its tempering stability changes with the quenching temperature (Figure 4b). At the quenching at 1030 °C, the tempering stability of Dievar-N steel is worse than that of Dievar steel. However, Dievar-N steel has higher tempering stability in the quenching temperature range of 1060–1100 °C. As the tempering time increases, the difference in tempering stability of hot-working die steel becomes increasingly obvious. At 1030 °C, most of V(C,N) is undissolved. Therefore, the contents of V and N involved in the precipitates of M_3_C in the matrix decrease, thereby reducing the tempering stability of the steel. Gu et al. [76] conducted an in-depth study on how trace N affects the microstructure evolution during long-term tempering. Figure 4c–f shows the transmission electron microscopy (TEM) images for the microstructure of two mold steels tempered at 600 °C for 48 h. It can be observed that after trace N alloying of Dievar steel, the precipitates of V(C,N) are promoted and the coarsening of M_23_C_6_ is inhibited. The presence of refined M_23_C_6_ particles distributed along the subgrain boundaries inhibits the mobility of these boundaries. Raising the quenching temperature increases the solid solubility of alloying elements while nitrogen (N) also influences the composition and nucleation rate of M_3_C carbides during the initial tempering stage. Consequently, this hinders the transformation and coarsening of carbides during subsequent long-term tempering processes. The above reasons collectively contribute to the effect of N on the tempering stability of die steel.

The trace N alloying of Cr-Mo-V series hot-working die steels can enhance their strength and toughness and improve the optimal quenching temperature range. In recent years, most new hot-working die steels are developed based on the Cr-Mo-V series; the addition of trace N can maximize the effect of V. Therefore, it is of great significance to investigate the trace N alloying of Cr-Mo-V series die steels for the development in high-performance hot-working die steels in the future.

### 3.3. Secondary Refinement and Purification by Electroslag Remelting

The size and distribution of carbides will greatly influence the performance of die steel [77]. Certain die steels encounter issues like segregation, resulting in coarse primary carbides distributed along dendritic boundaries, potentially acting as crack initiation sites. This result significantly influences the lifespan of the die steel [78]. Studies have shown that increasing the cooling rate can reduce solidification segregation, thereby refining primary carbides and improving their distribution and the strength and toughness of steel [79]. However, this method is not effective for large workpieces. The solidification rate of large ingots is difficult to control and cannot effectively tailor their microstructures. Forging can effectively refine the microstructures of steels, improving their density and mechanical properties [80].

Nevertheless, forging has limited influence on enhancing the purity of the steel and controlling the presence of inclusions within the ingot. Electroslag remelting (ESR) was developed rapidly in the 1960s as a secondary refining technology. Such a method can effectively control the inclusions, such as their size, quantity, shape, and distribution, thereby improving the solidification microstructure [64,81,82,83,84,85]. During the ESR process, deoxidation plays a crucial role as it effectively reduces the number of inclusions and hinders the formation of new ones.

**Figure 5:**
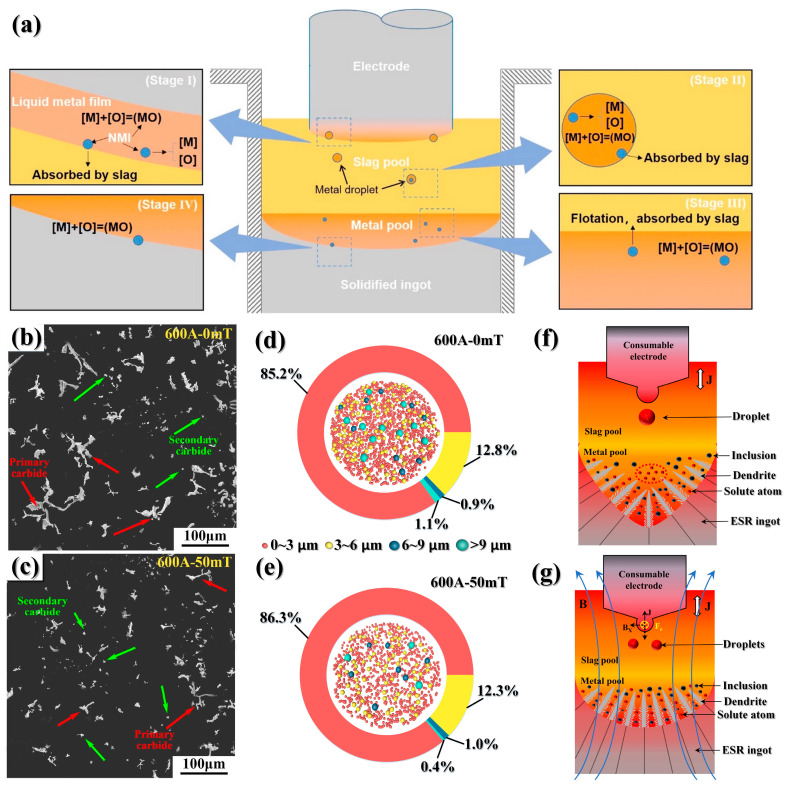
(**a**) Schematic illustration of inclusion removal and fresh inclusion generation during the ESR process [86]. Morphology of carbides in different ESR ingots: (**b**) 600 A; 0 mT, (**c**) 600 A; and 50 mT. Statistical chart of the inclusion percentage in ESR ingots: (**d**) 600 A; 0 mT and (**e**) 600 A; 50 mT. Mechanism diagram of the ESR process under different parameters: (**f**) absence of the ASMF treatment and (**g**) presence of the ASMF treatment [89].

Additionally, special attention must be given to the specific influence and interaction caused by the reoxidation of molten steel. Non-metallic inclusions are inevitable and cannot be eliminated. During the ESR process, new inclusions are also formed apart from the pre-existing inclusions that remain. Figure 5a shows the formation of non-metallic inclusions and the process of inclusion removal during ESR [86]. Stages Ⅰ, Ⅱ, and Ⅲ are oxidized inclusions generated inside the molten steel and stage Ⅳ entails new non-metallic inclusions generated during the solidification of molten steel. Therefore, the number of inclusions can only be minimized by regulating their size and shape and mitigating their influence on the material’s mechanical properties and service life.

ESR technology can meet the demand for high-quality hot-working die steel. However, as diverse requirements evolve, the traditional ESR technology has revealed various shortcomings. Researchers have made different improvements based on the traditional ESR technology according to different needs [87,88]. As the size of the die steel increases, the molten pool becomes deeper and the carbides would become coarser during the ESR process of large ingots. To solve such problems, Ma et al. [89] applied an axial static magnetic field (ASMF) to conventional ESR and analyzed the effect on H13 die steel. Figure 5b shows the carbide morphology of the traditional ESR process H13 die steel under a frequency of 50 Hz and 600 mA alternating current. It can be seen that the primary carbide microstructure is complex and coarse. As Figure 5c shows, after applying an axial static magnetic field of 50 mT, the type of carbides did not change. The primary carbides are refined and their shape changes into fine strips.

Figure 5d,e shows the number and percentage of inclusions without ASMF treatment and after ASMF treatment, respectively. It can be found that the large-size inclusions in the remelted die steel ingot are basically eliminated after ASMF treatment. Although the proportion of inclusions with certain sizes increases, the number of overall inclusions decreases, and large-size inclusions are absent. Figure 5f,g shows the solidification mechanism with or without ASMF treatment in the ESR process. After applying ASMF, the Lorentz force formed by the magnetic field and the alternating current leads to the dispersion and reduction in the size of the droplets. Therefore, the molten pool becomes shallow and the temperature and solute atoms in the molten pool are evenly distributed, which inhibits the segregation and refines precipitates. As the droplet size decreases, it takes longer to pass through the slag, resulting in improved removal of impurities and inclusions.

By incorporating ASFM into traditional ESR, the solutions to issues arising from expanding the molten pool in large die steel can be further enhanced. This process can refine the carbide, produce a more homogeneous composition, and more effectively eliminate inclusions. In order to improve their mechanical properties and produce high-quality and large-scale die steels, ESR technology was developed rapidly in the 1960s and 1970s. Technologies such as electroslag continuous casting, protective atmosphere ESR, high-voltage ESR, vacuum ESR, directional solidification, conductive casting, and external magnetic fields have been developed and continuously improved. As a result, ESR technology has become highly mature and extensively applied in the production of diverse steel materials.

### 3.4. Refined and Densified Microstructure via a Large Forging Ratio

Forging is an important process in the production process of die steel. It can refine the carbide and matrix microstructure and eliminate defects in the as-cast steel. As a result, the properties of the material, such as strength, toughness, fatigue resistance, and wear resistance, can be enhanced [90]. Numerous process parameters influence the forging effect, with the forging ratio, the ratio of the cross-sectional area of a metal before and after deformation, being one of the most crucial parameters [91]. A large forging ratio produces high-quality forgings, producing a fine, uniform, and dense microstructure. In turn, such a process enhances the mechanical properties of the die steel and establishes a solid foundation for subsequent heat treatment processes. However, the forging ratio should be well controlled.

On the one hand, a large forging ratio will increase the manufacturing cost. On the other hand, if the forging ratio is too large, the microstructures of die steels may become fibrous, reducing their performance. The microstructures and mechanical properties of steels with different compositions are consistent with the change in the forging ratio. The following two steels are taken as examples to illustrate the basic principle.

**Figure 6:**
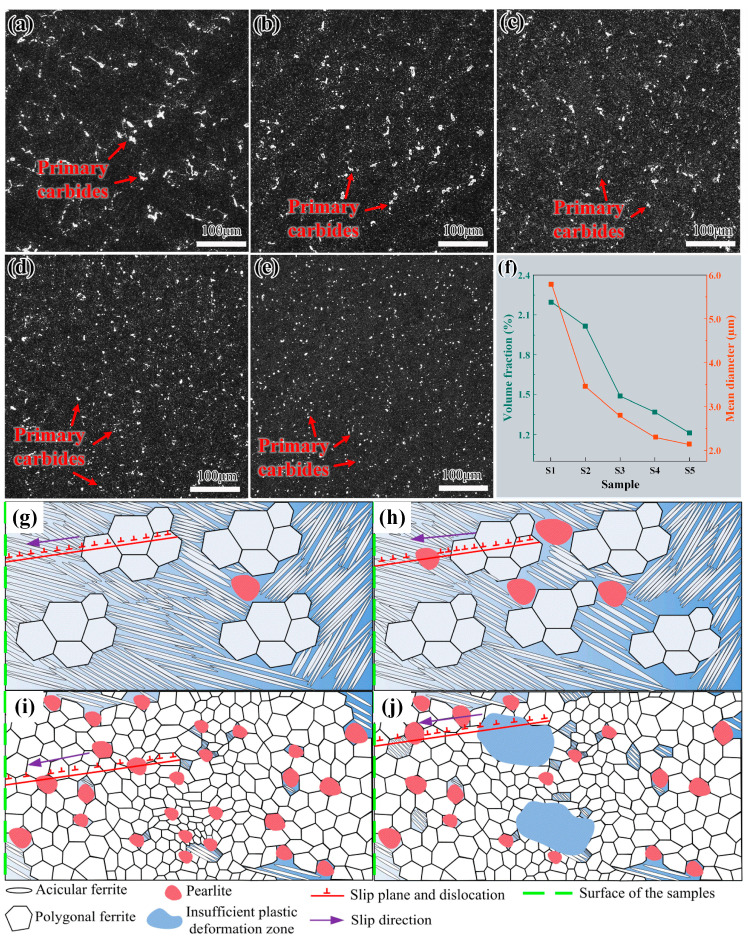
Primary carbide distribution of steels with different forging ratios: (**a**) S1; (**b**) S2; (**c**) S3; (**d**) S4; (**e**) S5; and (**f**) carbide volume fraction and mean diameter of S1–S5 [92], Dislocation slip under different forging ratios: (**g**) 2; (**h**) 3; (**i**) 4; and (**j**) 5 [93].

Jiang et al. [92] investigated the effect of different forging ratios on a new type of C-Cr-Mo-V martensitic steel. In Figure 6, the trend in the optical microstructure, volume percentage of primary carbides, and average diameter in the steel are depicted after varying forging ratios and subsequent quenching and tempering treatments. In Figure 6a,e, steels are marked as S1, S2, S3, S4, and S5, respectively, and the corresponding forging ratios are 0, 6, 8, 10, and 12. A correlation between the forging ratio and the size of primary carbides in the steel is revealed. Specifically, an increase in the forging ratio led to a gradual decrease in the size of primary carbides, resulting in a more uniform distribution. Figure 6f shows that the volume percentage of primary carbides also decreases with the increase in the forging ratio. Subsequently, the hardness and toughness of the steel were examined and it was found that the hardness and toughness increase with the increase in the forging ratio and the strengthening is mainly attributed to the refinement of the microstructure. In the wear test, the relationship between the wear mass loss and the forging ratio is not linear. The wear resistance is improved when the forging ratio is 6 and 12. When the forging ratio is 6, the primary carbide content is reduced. However, the carbides are refined and evenly distributed. When the forging ratio is 12, many secondary carbides precipitate in the matrix after heat treatment. The primary carbides are refined further and the martensitic lath is refined. Therefore, a large forging ratio can improve the wear resistance of steel.

Dai et al. [93] investigated the ability of steels to resist fatigue crack growth at different forging ratios. Figure 6g,j is a schematic diagram of dislocation slip in steel under different forging ratios. At a forging ratio of two, dislocations tend to pass through the acicular ferrite, whereas the pearlite cluster inhibits the dislocation movement when the forging ratio increases to three (Figure 6g,h). Hence, the resistance to crack extension is enhanced. When the forging ratio reaches four and five, the grains are significantly refined, hindering the dislocation movement. As such, the crack resistance is further improved.

Nevertheless, as the forging ratio reaches five, the fatigue crack resistance of the forged steel may be adversely affected due to the insufficient plastic deformation zone. Therefore, when the forging ratio is four, the fatigue resistance of the steel is relatively excellent. The application of a substantial forging ratio stands out as the foremost determinant for achieving high-quality forgings. It amplifies the grain refinement effect and contributes to the enhancement of mechanical properties such as hardness, toughness, and wear resistance. Extensive research conducted by predecessors has effectively established the significance of the forging ratio, incorporating it as a well-developed component within the steel production process. Consequently, the current research on the forging ratio poses challenges in making substantial improvements or breakthroughs.

### 3.5. Optimization of Heat Treatment

The main reason for the failure of die-casting molds is the improper heat treatment process. Therefore, optimizing the heat treatment process design for mold steel (as well as other metallic materials) becomes a crucial approach to prevent premature steel failure and enhance the lifespan of molds [32,94,95,96]. The primary objective of heat treatment is to enhance the mechanical properties of steel by modifying either its microstructure or the chemical composition of the surface. This process is accomplished through heating, heat preservation, and cooling, occasionally excluding the heat preservation stage [97,98]. Due to the intricate nature of steel microstructures, many heat treatment procedures are available [99,100,101]. Heat treatment is also an indispensable process for producing hot-working die steels. Different grades and different applications of die steels require different heat treatment processes. Even for the same steel, different heat treatment processes will have different outcomes [102,103,104]. With the integrated die-casting trend, many new hot-working die steels have been emerging consistently. Consequently, the design and optimization of their heat treatment processes have become increasingly crucial.

The heat treatment process can be divided into two categories: overall heat treatment and surface heat treatment. The overall heat treatment is the most important for die steels. The focus of the overall heat treatment lies in the selection of the heating temperature, holding time, and cooling method to achieve the optimal steel performance [105,106,107]. It has long been discovered that the properties of steel are affected by changes in temperature. After a long period, the optimized heat treatment process is developed. To establish the heat treatment process for a novel hot-working die steel, the procedure typically involves the following steps: initially, employing the thermal expansion method or thermal analysis approach to determine the phase transition temperature of the die steel, which serves as a reference for subsequent heat treatment processes; next, selecting an appropriate annealing process based on the steel’s alloy element content and specific performance requirements; and finally, conducting an orthogonal test analysis on the quenching and tempering process of the annealed steel to identify the optimal temperature range and holding time that yields superior performance.

**Figure 7:**
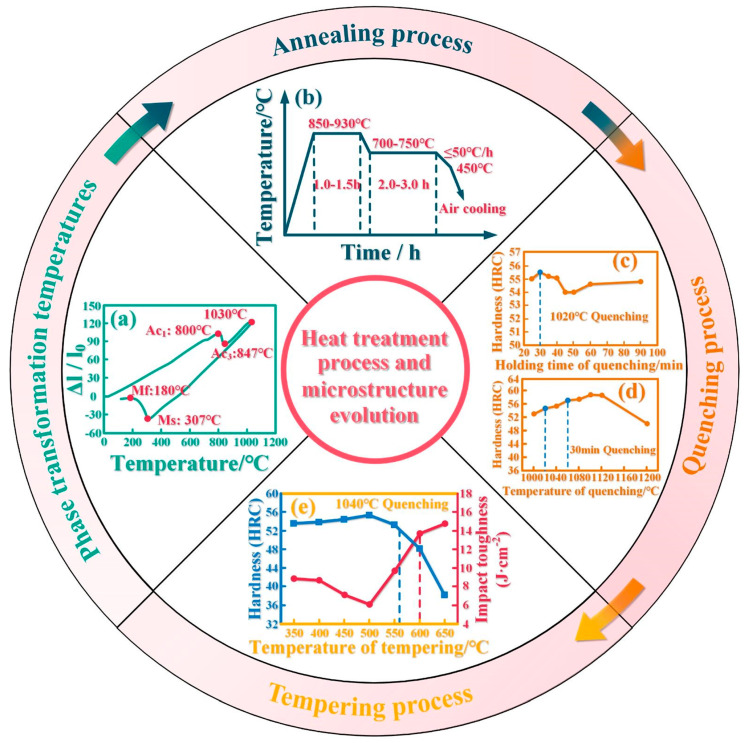
Heat treatment process design process and microstructure evolution diagram of new nitrogen-containing hot-working die steel: (**a**) phase transformation temperatures; (**b**) annealing process; (**c**) and (**d**) quenching process; and (**e**) tempering process [108].

**Figure 8:**
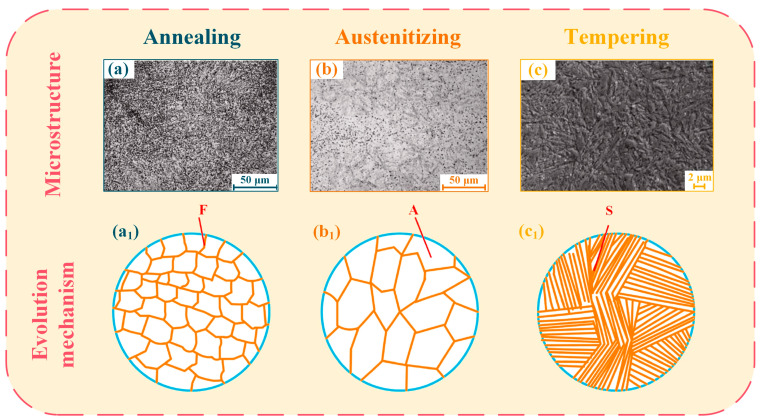
Microstructure and evolution mechanism diagrams of H13 steel after different heat treatments: (**a**,**a_1_**) annealing process; (**b**,**b_1_**) austenitizing process; and (**c**,**c_1_**) tempering process [108,109,110].

A new hot-working die steel requires a corresponding heat treatment optimization to achieve the best comprehensive mechanical properties. Figure 7 is a heat treatment process design flow chart of a new N-containing die steel based on H13 steel [108]. Figure 8 shows the microstructure and evolution mechanism diagram of H13 steel at different stages in the heat treatment process [108,109,110]. First, the phase transformation temperature of the new N-containing die steel is measured by the linear expansion method (Figure 7a). The austenite phase transformation critical temperatures (Ac1 and Ac3) are 800 °C and 847 °C, respectively. The martensite phase transformation critical temperatures (Ms and Mf) are 307 °C and 180 °C. These phase transformation temperatures are important for subsequent heat treatment process design. After forging, die steels usually need to be annealed as a preheating treatment to achieve the purpose of stress relief and work hardening so that their compositions and microstructures are uniform and can be prepared for the subsequent quenching treatment [111]. Based on the alloying element composition, it can be determined that the die steel is hypereutectoid. Considering the processing performance and other factors, the ultimate decision is to utilize isothermal spheroidizing annealing.

The annealing process of new N-containing hot-working die steel is shown in Figure 7b. Spheroidal pearlite with a uniform microstructure would be obtained after spheroidizing annealing (Figure 8a,a_1_). Spheroidizing annealing is often used as an annealing process for hot-working die steels [103]. Corresponding annealing methods can also be used for steels with special requirements. For example, complete annealing or isothermal annealing is usually used for hypoeutectoid steels [112]; recrystallization annealing can be used for steels with severe work hardening [113,114]; stress relief annealing can be used for steels with excessive internal stress [115,116]; and diffusion annealing is frequently employed for steels exhibiting significant segregation or demanding high levels of microstructural and compositional uniformity [117].

The final heat treatment for die steel involves quenching and tempering. To simultaneously achieve the desired strength and toughness for die steel, a common approach is to combine both heat treatments [118,119]. Li et al. [108] found the optimal quenching and holding time for die steel at a quenching temperature of 1020 °C. It can be seen that the hardness of die steel is the highest when holding for 30 min (as shown in Figure 7c). Subsequently, the quenching temperature was altered while maintaining a 30-min quenching duration. It was observed that the hardness of the die steel consistently rose with increasing quenching temperature. However, a rapid decline in hardness occurred when the temperature was elevated from 1120 °C to 1200 °C (Figure 7d). According to the actual demand for the die steel, it can be determined that the optimum quenching temperature range is 1020–1060 °C. The quenched microstructure after austenitizing consists of martensite, retained austenite, and dispersed carbides (Figure 8b,b_1_). Hot-working die steel has high hardenability requirements, so oil cooling or air cooling is frequently used. In comparison, water cooling generally aims at metals with weak hardnesses, such as low-carbon steels [120,121].

Finally, it is necessary to determine the ideal tempering temperature and time for mold steel. Generally, the tempering time ranges from 1 to 3 h and the specific duration can be determined based on the thickness of the die steel and tempering conditions. The newly developed die steel undergoes a 2-h tempering process, after which its hardness and impact toughness are tested across various temperatures. The hardness and toughness show an opposite trend with the increase in the tempering temperature and the overall performance of die steel is optimal in the tempering temperature range of 560–600 °C (as shown in Figure 7e). The tempered microstructure after quenching is shown in Figure 8c,c_1_, which is mainly composed of tempered sorbite and a small number of carbides.

After conducting a series of exploratory tests, it has been determined that the heat treatment process for this new nitrogen-containing hot-working die steel is as follows: an isothermal spheroidizing annealing process is employed for annealing; quenching is performed at a temperature range of 1020–1060 °C for 30 min; and tempering is carried out at a temperature range of 560–600 °C for 2 h. The above is the basic heat treatment process of a newly developed hot-working die steel. So far, industrialized high-quality die steel uses vacuum heat treatment. Therefore, it can be further optimized according to specific requirements, such as multiple tempering and surface heat treatment [122,123]. There are many heat treatment processes and these processes are interrelated. Hence, it is imperative to design experiments based on the specific characteristics of the die steel and its intended application to continually explore the optimal heat treatment process. This process ensures that the new hot-working die steel can fully exhibit its outstanding overall performance.

## 4. Nanoparticle Reinforcements

Currently, most traditional methods, such as melt purification, large forging ratios, and heat treatment optimization, have reached a bottleneck, making it challenging to significantly enhance the overall performance of steel. Under the general trend in integrated and large-scale mold, higher requirements are put forward for the performance of die steel. Hence, there is an urgent need to develop a new, high-efficiency, cost-effective, and environmentally friendly process to enhance the performance of die steel, enabling it to meet the new requirements.

Adding trace nano-ceramic particles into the matrix has been proven to improve the strength and toughness of materials simultaneously [110,124,125,126,127,128,129], which is beneficial to control the uniformity of microstructure and composition of large-size ingots and solves the problem of uneven performance in various regions of large-size die steel. Furthermore, the comprehensive performance of die steel based on traditional strengthening methods can be improved. Additionally, it can synergistically collaborate with the conventional strengthening process, presenting a novel, cost-effective, and highly efficient method of reinforcement [130]. The ceramic particles that strengthen the steel matrix are mainly divided into carbides, borides, oxides, and nitrides [131]. Commonly used ceramic particles include TiC, TiB_2_, Al_2_O_3_, TiN, etc. [110,132,133]. Among them, TiC and TiB_2_ particles have extremely high melting points, hardness, and good chemical stability and both ceramic particles have a good lattice-matching relationship with the main target phase, making them outstanding reinforcements for steel [134,135,136]. There are ex situ and in situ methods for adding ceramic particles to the steel matrix. The ex situ method involves direct contact between ceramic particles and molten steel. While this approach offers convenient additions, it also introduces several challenges and issues. First, there may be a large difference in specific gravity between ceramic particles and molten steel, which poses a considerable challenge in achieving successful particle addition. Second, nanoparticles are easy to agglomerate in molten steel and it is difficult to disperse them uniformly.

Moreover, ceramic particles are also easy to cause surface contamination. The advantages of the in situ method over the ex situ method are apparent. The in situ synthesized ceramic particles have no pollution on the steel surface and have good wettability with the matrix [137]. Corresponding solutions to various problems of ceramic particles have been developed in recent decades [138,139,140]. However, there is still a lack of an innovative processes that can solve all problems simultaneously.

**Figure 9:**
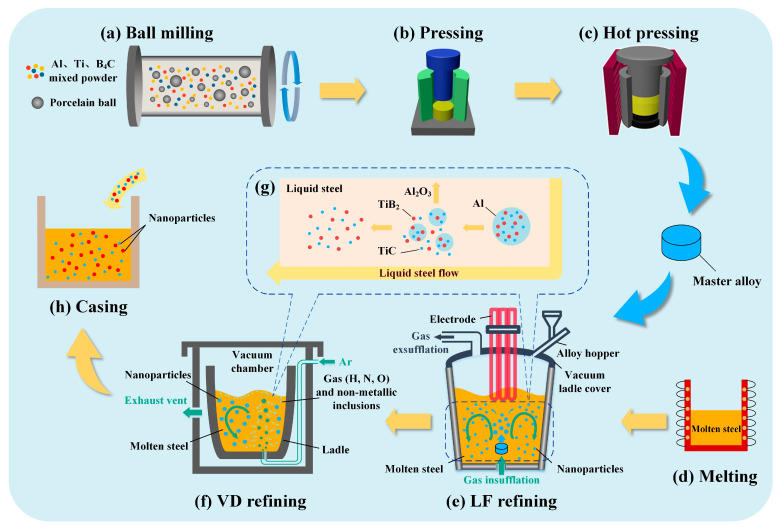
Flow diagram of the preparation of the in situ TiC-TiB_2_ duplex ceramic nanoparticles’ reinforced steel and a schematic diagram of the dispersion of nanoparticles in the melt [49,141,142,143].

Figure 9 is the preparation process of in situ TiC-TiB_2_ dual-phase nano-ceramic particle-reinforced steel. In recent years, based on the self-propagating high-temperature synthesis (SHS), the research group has used the prefabricated aluminum-based nano-ceramic particle master alloy as the carrier; the TiC-TiB_2_/Al master and TiC/Al master alloy were successfully prepared [125,126,127,128]. The TiC-TiB_2_/Al master alloy is prepared from the Al-Ti-B4C system. The Al powder, Ti powder, and B4C powder are mixed according to a certain ratio and then milled in a high-energy ball mill (Figure 9a). Finally, the master alloy can be obtained by cold pressing and hot pressing in sequence (Figure 9b,c) [141]. The nanoparticles in the ladle are released into the molten steel along with the melting of the Al matrix of the intermediate alloy. Subsequently, the Ladle Furnace (L.F.) refining and Vacuum Degassing (V.D.) are required to remove impurities in the sequence during the production process (Figure 9e,f). The blown Ar bubbles make the molten steel fully convected and stirred and the molten steel rolls up and down to continuously release and evenly disperse the nanoparticles in the molten steel. The Al matrix also plays a role in deoxidation and floats to the surface of molten steel in the form of alumina. The schematic diagram of the dispersion of nanoparticles is shown in Figure 9g. It satisfies the uniform dispersion of nanoparticles and plays a role in purifying the melt.

Moreover, using the Al-based master alloy as the carrier also solves the problem; the nanoparticles can easily float during the L.F. and V.D. processes. Finally, the casting process yields an ingot of steel reinforced with nanoparticles. This preparation process effectively addresses the challenges associated with nanoparticle introduction, propensity for agglomeration, and surface contamination. In the previous work, adding TiC ceramic particles as a single-phase reinforcement to the steel matrix can strengthen the steel [140,141,142,143]. The simultaneous addition of TiC and TiB_2_ to the steel matrix revealed that the TiC-TiB_2_ dual-phase ceramic particles significantly influence the overall performance. The strengthening effect of TiC-TiB_2_ dual-phase ceramic particles is more significant than that of TiC single-phase nanoparticles [103,135]. Therefore, the strengthening mechanism of H13 steel strengthened by trace TiC-TiB_2_ dual-phase nano-ceramic particles is taken as an example below.

**Figure 10:**
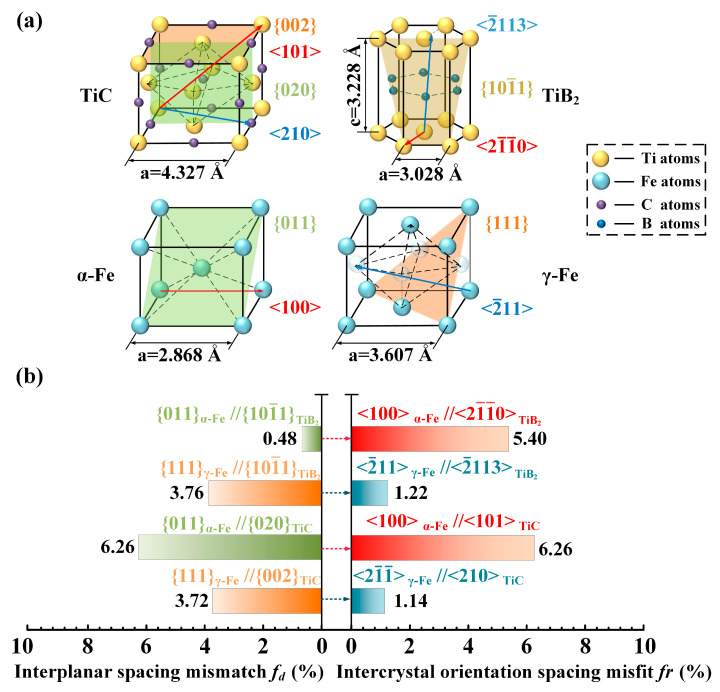
(**a**) Schematic diagram of the crystal structure, closest-packed crystal orientation, and closest-packed crystal plane of TiC, TiB_2_, and α-Fe, γ-Fe. (**b**) Crystal structure of TiC, TiB_2_, and α-Fe; γ-Fe interplanar and intercrystal orientation spacing mismatch butterfly diagrams [109,142,144].

First, Qiu et al. [109,142] analyzed the lattice mismatch relationship between nano-TiC-TiB_2_ particles and the main target phases in H13 steel. Table 3 shows the interplanar spacing mismatch and intercrystal orientation spacing mismatch obtained after theoretical calculations using the Edge-to-Edge Matching (E2EM) model. The crystal structures, closest-packed crystal planes (C.P. plane), and closest-packed crystal orientations (C.P. orientation) of ceramic particles and α-Fe and γ-Fe are shown in Figure 10a. Figure 10b uses the butterfly diagram to more intuitively show the interplanar mismatch and intercrystal orientation spacing mismatch [109,142,144]. The mismatches between TiC and TiB_2_ particles with α-Fe and γ-Fe phases are less than 10%. This phenomenon indicates a strong integration of the two nano-ceramic particles with the γ-Fe and α-Fe phase interfaces, making them suitable as heterogeneous nucleation cores for γ-Fe and α-Fe during the solidification of H13 steel.

**Figure 11:**
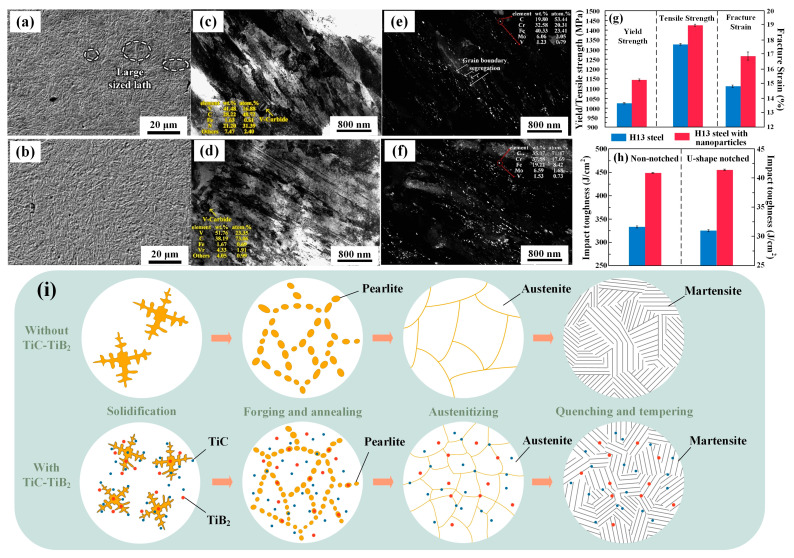
Comparison of microstructure, properties, and mechanism of H13 and H13-reinforced steels. SEM image: (**a**) without nanoparticles and (**b**) with nanoparticles. TEM bright field image: (**c**) without nanoparticles and (**d**) with nanoparticles. TEM dark field image: (**e**) without nanoparticles and (**f**) with nanoparticles. Histogram of mechanical properties: (**g**) comparison of tensile properties, (**h**) comparison of impact properties, and (**i**) comparison of microstructure evolution [109,142].

Figure 11 compares the microstructure, mechanical properties, and microstructure evolution between no nanoparticles and 0.02 wt.% TiC-TiB_2_ nanoparticles added [109,142]. The nanoparticle content of 0.02 wt.% was chosen because exploratory experiments have revealed that increasing the trace nanoparticle content results in improved performance of the nanoparticle-reinforced steel. However, when the content of added particles reaches 0.03 wt.%, the performance of reinforced steel becomes unstable. Hence, the reinforced steel with 0.02 wt.% nanoparticle is finally selected for comparison with the base steel [143]. Figure 11a,b shows SEM images for the microstructures of H13 steel and H13 nanoparticle-reinforced steel after heat treatment. The microstructures of both steels are mainly composed of lath-shaped tempered martensite. However, the martensite lath in H13 steel is thick, which will have a detrimental influence on the mechanical performance of the steel. The addition of nanoparticles results in the refinement of large-sized martensitic laths and a more compact microstructure. These changes simultaneously enhance the strength and toughness of H13 steel.

The size and distribution of carbides in H13 steel and H13 nanoparticle-reinforced steel are analyzed by TEM images (Figure 11c–f). Furthermore, the addition of nanoparticles results in lower martensitic laths, as depicted in the bright field images of Figure 11c,d. Based on the dark field images shown in Figure 11e,f, it is evident that the distribution of carbides in H13 steel is uneven. Some carbides are segregated along the grain boundaries, while larger-sized carbides precipitate within the grains. In comparison, the segregation phenomenon of carbides in H13 reinforced steel is weakened; their distribution is more uniform and their size is also reduced. Figure 11g,h shows the test results of tensile and impact properties. The addition of nanoparticles leads to significant improvements in the yield strength, tensile strength, fracture strain, and impact toughness of H13 steel, with varying degrees of enhancement. Such an outcome signifies a breakthrough and pioneering innovation, particularly when traditional strengthening processes have been predominantly hindered. As a result, a new pathway has been paved for advancing high-performance die steel.

The microstructure evolution of H13 steel, strengthened by nano-TiC-TiB_2_ dual-phase ceramic particles, is shown in Figure 11i. TiC-TiB_2_ particles are important in the solidification, forging, and heat treatment stages. In the solidification stage, nanoparticles can be used as the nucleation sites due to the low degree of mismatch with α-Fe and γ-Fe. Nanoparticles also hinder the dendrite growth at the grain boundaries, inhibiting micro-segregation. By incorporating nanoparticles, the nucleation temperature can be increased and heterogeneous nucleation can be facilitated. As a result, solidification becomes more uniform, leading to a refined microstructure. Throughout the forging and annealing stages, the grain refinement during solidification is preserved, alongside recovery and dynamic recrystallization. In this process, the nanoparticles serve as nucleation sites for austenite. In the austenitization stage, the nanoparticles at the grain boundaries hinder grain growth due to the pinning effect and inhibit carbide segregation. In the quenching and tempering stage, nanoparticles, combined with the established legacy of refined austenite grains, contribute to the refinement and increased quantity of martensite laths during phase transformation. The fine carbides are dispersed on the martensitic grain boundaries, further tailoring the martensite microstructure. As stated above, by introducing nanoparticles, the microstructure is refined and the segregation of carbides is effectively inhibited. As a result, H13-reinforced steel exhibits outstanding comprehensive properties and an extended service life.

The addition of nanoparticles has significantly enhanced the strength and toughness of H13 steel. The strengthening and toughening mechanisms of nanoparticle-reinforced steel are analyzed based on the evolution of its microstructure. Including nanoparticles promotes heterogeneous nucleation, inhibits grain growth, and refines the grains. More numerous and finer grains help to mitigate stress concentration, increase the grain boundary area, and enhance the resistance to crack propagation of steel. As a result, the strength and toughness of H13 steel have been greatly improved through refinement strengthening. Due to the different thermal expansion coefficients of the nanoparticles and the steel matrix, the temperature change during solidification and heat treatment would generate thermal mismatch stress. Increased dislocation density plays a strengthening role (thermal mismatch strengthening) in enhancing the strength of steel. During plastic deformation, the movement of dislocations will be inhibited by the highly dispersed Cr_2_C_3_ precipitates in the steel. As the dislocation lines bypass the second phase, the formation of dislocation loops and their subsequent proliferation occurs. Consequently, the dislocation density experiences a significant increase, leading to intensified interactions between dislocations. This results in the further improvement in strength of H13 steel.

The introduction of a pre-dispersed and endogenous nano-particle Al-based master alloy into the molten steel serves as a successful carrier for achieving the uniform dispersion of nanoparticles throughout the steel. This process enables the successful preparation of H13 steel reinforced with a small amount of dual-phase nano-TiC-TiB_2_ particles. This innovative method offers several advantages, including low cost, high efficiency, and no pollution. It can effectively meet the demands for high-quality steel in the new era, providing a new avenue for further enhancing steel strengthening techniques. In addition, there are new steel fabrication processes without requiring mixing by melting, such as the steel mechanosynthesis method [145]. As well as environmentally friendly methods for the production of metals or nanoparticles, such as the low-energy magnetically-driven milling technique [146], this method may become a more optimal solution for the preparation of the nanoparticles described above. Hopefully, it will give more inspiration to researchers in related fields.

## 5. Summary and Prospects

In recent years, the issue of global warming has become intensified significantly. The automotive industry is faced with the imperative of conserving energy and reducing carbon emissions to achieve sustainable development. To accomplish this objective, reducing the weight of the car proves to be an exceptionally effective method. Moreover, lightweight design is currently a principal focus for many car companies. Once Tesla proposed integrated die-casting technology, it became a disruptive automobile manufacturing technology. Integrated die-casting technology replaces multiple scattered parts with highly integrated casting parts, eliminating the need for cumbersome stamping and welding processes and enabling one-time molding. This technology not only saves on cost but also achieves vehicle lightweighting. Various countries are actively following up on this innovative technology. Integrated die-casting technology has become a general trend. Subsequently, owing to the necessity of large and intricate molds in integrated die-casting technology, the requirements for the performance of die steel are raised significantly, surpassing the capabilities of conventional die steel to meet such demanding requirements.

This article reviews the new hot-working die steel and strengthening technology. One of the important ways is to optimize the alloy composition to develop new high-performance die steel. Another important way is to develop or optimize the production process and heat treatment process of die steel. The heat treatment process has reached a high level of maturity, with a relatively systematic exploration of process parameters. The traditional production process mainly includes melt purification, alloying, large forging ratio, and electroslag remelting. In recent years, making breakthroughs in these processes and achieving significant improvements in the performance of die steel and its industrial application have proven to be challenging.

Nanoparticle-reinforced technology can significantly improve the strength and toughness of die steel simultaneously. However, due to the difficulty in adding nanoparticles, easy agglomeration in the melt, uneven dispersion, and surface pollution limit nanoparticle-reinforced technology development and application. Fortunately, based on self-propagating high-temperature synthesis (SHS) technology and prefabricated Al-based nano-ceramic particle master alloy (carrier), the TiC/Al master alloy and TiC + TiB_2_/Al master alloy can be successfully prepared by the nanoparticle-reinforced technology. As a result of the in situ method of adding nanoparticles, the problem of being easily polluted or oxidized by the prepared die steel is avoided. The wettability of the master alloy and the steel matrix is also good. This breakthrough has significantly enhanced the overall performance of die steel and prolonged its lifespan. The affordability, stability, and outstanding comprehensive performance of this technology have paved the way for its widespread application. At present, the nanoparticles-strengthened hot-working die steel has been industrialized. Moreover, nanoparticle-strengthening technology has also achieved remarkable results in the comprehensive performance enhancement of other types of steel, such as stainless steel, bearing steel, carbon structural steel, and medium carbon modulated steel.

After undergoing extensive development, the traditional strengthening technology has reached a state of perfection and maturity. How to overcome the shortcomings of the new strengthening technology and realize its industrialization is a difficult problem that urgently needs to be broken through. The development in new die steels and how to better combine the new strengthening technology with the traditional strengthening technology are also the focus of future research. This article is anticipated to offer novel insights to researchers in relevant fields, enabling them to enhance or develop efficient technologies for strengthening die steel. This, in turn, will facilitate meeting the demands of the new era of integrated die-casting more effectively.

## Figures and Tables

**Table 1:** Commonly used international grades of hot-working die steels. GB is the National Standard of the People’s Republic of China; ASTM is the American Society of Testing Materials Standard; AISI is the American Iron and Steel Institute Standard; JIS is the Japan Industrial Standard; DIN is the German Industry Standard; and ISO is the International Organization for Standardization.

GB	ASTM/AISI	JIS	DIN	ISO
5CrMnMo	-	SKT3	-	-
5CrNiMo	L6	-	1.2714	-
5CrNi2MoV	-	SKT4	-	55NiCrMoV7
-	6G	SKT5	1.2323	-
4CrNi4Mo	-	SKT6	-	45NiCrMo1-6
4CrW2Si	S1	SKS41	1.2542	45WCrV2
4Cr3Mo3SiV	H10	SKD7	1.2365	X32CrMoV3-3
4Cr5MoSiV	H11	SKD6	1.2343	X37CrMoV5-1
4Cr5MoSiV1	H13	SKD61	1.2344	X40CrMoV5-1
3Cr2W8V	H21	SKD5	1.2581	X30WCrV9-3

**Table 3:** Calculation results of interplanar spacing mismatch (ƒd) and intercrystal orientation spacing mismatch (ƒr). C.P. plane is the closest-packed crystal planes and C.P. orientation is the closest-packed crystal orientations.

Phase	Crystal Structure	Lattice Parameter/nm	C.P. Plane	ƒd	C.P. Orientation	ƒr	Ref.
γ-Fe	FCC	a = 0.3618	{111}	3.72%	<$2\bar{1}\bar{1}$>	1.14%	[142]
TiC	FCC	a = 0.4327	{002}		<210>		
α-Fe	BCC	a = 0.2866	{011}	6.26%	<100>	6.26%	[143]
TiC	FCC	a = 0.4327	{020}		<101>		
γ-Fe	FCC	a = 0.3618	{111}	3.76%	<$\bar{2}$11>	1.22%	[109]
TiB_2_	HCP	a = 0.3028	{$10\bar{1}$1}		<$\bar{2}$113>		
		c = 0.3228					
α-Fe	BCC	a = 0.2866	{011}	0.48%	<100>	5.40%	[109]
TiB_2_	HCP	a = 0.3028	{$10\bar{1}$1}		<$2\bar{1}\bar{1}$0>		
		c = 0.3228					

## Data Availability

Not applicable.
